# Effects of Oat Complex High-Fiber Formula Powder on the Composition of Intestinal Microbiota and Enzyme Activities in Mice Induced by a High-Fat Diet

**DOI:** 10.3389/fnut.2022.871556

**Published:** 2022-05-24

**Authors:** Rui Huo, Meili Zhang, Yakun Zhang, Xue Bai, Yuanyuan Zhang, Xinyue Guo

**Affiliations:** College of Food Science and Engineering, Inner Mongolia Agricultural University, Hohhot, China

**Keywords:** oat, formula powder, dietary fiber, intestinal flora, lipid metabolism, AMPK

## Abstract

Using oat-corn-konjac extruded mixed powder, oat bran micro powder, skim milk powder, Pueraria whole powder, and pumpkin powder as raw materials, a formula powder with high dietary fiber was prepared, and its effect on obesity in mice with a high-fat diet was investigated. After 7 days of adaptive feeding, the mice were divided into blank group, high-fat diet group, formula powder + high-fat diet group, and weight-loss drug + high-fat diet group. After 8 weeks of treatment, the body weight of mice were observed and measured to determine the composition of tract flora, liver leptin content, insulin content, and activities of AMP-activated protein kinase (AMPK), lipoprotein lipase (LPL), fatty acid synthetase (FAS), sterol-regulatory element-binding proteins (SREBPs), and acetyl CoA carboxylase 1 (ACC1). The results indicated that treatment with the formula powder could reduce the body weight of mice and increase the abundance of *Bifidobacterium, Akkermansia*, and *Romboutsia* compared to the group given a high-fat diet. Moreover, the leptin and insulin contents of the experimental group decreased from 5.67 μg/L to 0.12 μg/L and from 12.71 μg/L to 7.13 μg/L, respectively, compared to the control group, which was not significantly different from the blank group (P > 0.05). Also, the activities of AMPK and LPL increased, and the activities of FAS, SREBPs, and ACC1 were significantly decreased (P < 0.05). Some pathogenic bacteria were significantly positively correlated with leptin and FAS and significantly negatively correlated with LPL. Some beneficial bacteria were positively correlated with LPL. Therefore, the formula powder used in this study could reduce the body weight of mice, increase the abundance of some beneficial bacteria in the colonic intestinal microbiota, and improve the activities of enzymes related to lipid metabolism in the liver. This study provides a theoretical reference for the pathway by which high-fiber diet improves liver and intestinal metabolic abnormalities.

## Introduction

Meal-replacement powders are reconstituted products with the functional characteristics of convenience for eating and improvement of health (1, 2). Based on the raw materials, they may be classified into cereal meal-replacement powders, fruit-and-vegetable meal-replacement powders, protein meal-replacement powders, and dietary-fiber meal-replacement powders. Recently, the meal-replacement powder industry has been booming, and the differences among meal replacement powders are increasingly blurred. In product design, the meal-replacement powders are no longer purely composed of cereal powders, protein powders, or fruit-and-vegetable powders but comprise a mixture of these powders according to the requirement for balanced and comprehensive intakes, as well as the specific functions of various nutrients (3). Of these, complex formula powders with oats and their by-products as the main raw materials can reduce blood sugar and fat, have high solubility, and can promote weight loss (4), owing to which, they are blessed with a bright future.

Existing research has revealed that the proportions of slowly digestible starch and resistant starch are significantly increased, and the rate of *in vitro* digestion is remarkably decreased after the extrusion treatment of oat, corn, and konjac powders (5). Besides, konjac powder, which is a common ingredient in the dietary field, is rich in glucomannan and has diverse bio-active substances (6). Oat bran powder is rich in β-glucan and absorbs dietary cholesterol (7), while reducing the glucose concentration in the small intestine (8) to maintain intestinal homeostasis. Research has further pointed out that the water-solubility, water-absorbability, and the rate of dissolution of soluble dietary fiber are increased after the super-micro grinding of oat bran powder (9, 10), with a demonstration of the thickening and dispersing effects (11). Given these facts, the oat bran micro-powder was selected as the main component of the formula powder in this work. Pumpkin powder is rich in trace elements; cobalt is relatively abundant, which can distend the blood vessels and reduce the blood pressure during *in vivo* metabolism, while the pectin content of pumpkin has an extensively diverse molecular structure (12). This alleviates the catalysis of amylase and postpones the absorption of carbohydrates by the intestinal tract (13, 14) to prevent and treat diabetes. Pueraria is rich in puerarin and flavonoids that are beneficial to the human body (15, 16). As a novel product developed recently, the whole powder of Pueraria is rich in various active constituents other than starch and is characterized by the requirement of simple processing techniques, the high utilization ratio of raw materials, and low production costs of the final product (17). The formula powder prepared using the above raw materials complies with the requirements of customers, such as color, taste, and stability in solution, and is consistent with the concept of healthy weight loss owing to its diverse functional ingredients and high satiety value. In this research, the effects of the proposed formula powder on obesity in mice with a high-fat diet were investigated to provide reference data for the development and utilization of cereal complex formula powders.

## Materials and Methods

### Materials and Reagents

In this study, 50 SPF healthy male C57BL/6J mice aged 6-weeks and bodyweight of (20 ± 2) g were purchased from the Animal Test Center, Inner Mongolia Medical University. The low-fat feed (with 10% of the energy coming from fat) and high-fat feed (with 60% of the energy coming from fat) were purchased from SPF (Beijing) Biotechnology Co., Ltd. The meal-replacement powder solution (with a mass ratio of 2.65 g/kg and concentration of 23%) was prepared, while Orlistat capsules (with a mass ratio of 60 mg/kg) were purchased from Zhongshan Wanhan Pharmaceutical Co., Ltd.

Meanwhile, oat bran powder was purchased from Inner Mongolia Xibei Huitong Agricultural Science and Technology Development Co., Ltd. Oat powder was purchased from Inner Mongolia Yitai Ecological Agricultural Co., Ltd. Dent corn was obtained from the Corn Research Center, Inner Mongolia Agricultural University. The J08 konjac powder was purchased from Hubei Konson Konjac Gum Co., Ltd. Skim milk powder was procured from Fonterra Co-operative Group. Pumpkin powder was obtained from Xinghua Lvshuai Food Co., Ltd. Pueraria whole powder was purchased from Pingle Leyao Food Co., Ltd., and silicon dioxide was purchased from Shandong Yunzhiran Biotech Co., Ltd.

Besides, ELISA kits for mouse AMP-activated protein kinase (AMPK), mouse fatty acid synthetase (FAS), mouse lipoprotein lipase (LPL), mouse sterol-regulatory element-binding proteins (SREBPs), mouse acetyl-CoA carboxylase 1 (ACC1), mouse leptin (LEP), and mouse insulin were purchased from Wuhan Xinqidi Biotech Co., Ltd., while the E.Z.N.A.® soil DNA extraction kit was purchased from Omega Bio-Tek.

### Instruments and Devices

For this study, an S32 test-type twin-screw extruder was purchased from Jinan Saixin Machinery Co., Ltd. A PLS new-type super micro grinder was purchased from Jinan Pulaishen Machinery Equipment Co., Ltd. A Chemray 800 fully-automatic biochemical analyzer was obtained from Shenzhen Rayto Biotech Co., Ltd. A KZ-II high-throughput tissue grinder was procured from Wuhan Servicebio Technology Co., Ltd. A SynergyH1 multi-mode microplate reader was purchased from BioTek Instruments, Inc.

### Test Method

#### Preparation of the Formula Powder

For the preparation of the oat-corn-konjac extruded mixed powder, the oat powder and corn powder were first separately sifted with a 60 mesh/inch sieve and then mixed according to the designed mixing ratio. Then, an appropriate amount of konjac powder was added and stirred thoroughly, following which, an appropriate amount of water was added and blended. Finally, the mixture was placed into the inlet of a twin-screw extruder with an extrusion temperature of 180°C, feed rate of 15 Hz, and screw speed of 16 Hz. For the preparation of the oat bran micro powder, 200 g of coarse oat bran powder was first weighed, ground with a super micro grinder, and then cured in a microwave oven at a microwave frequency of 800 W and for a curing time of 100 s. These two powders were then mixed with the skim milk powder, Pueraria powder, and pumpkin powder, according to the designed mixing ratio to yield the corresponding formula powder (Table 1).

**Table 1:** Nutritional composition of meal-replacement powder.

**Total starch**	**Total dietary**	**Soluble dietary**	**Insoluble dietary**	**Protein**	**Fat**	**Ash**	**Polyphenol**
**content/%**	**fiber content/%**	**fiber content/%**	**fiber content/%**	**content/%**	**content/%**	**content/%**	**content/%**
47.33 ± 5.10	19.31 ± 1.34	7.9 ± 0.65	11.42 ± 0.98	15.93 ± 1.05	6.56 ± 0.84	3.49 ± 0.38	0.19 ± 0.01

#### Animal Rearing Conditions and Grouping

After preparatory feeding for a week, the mice were arranged using a random allocation method into four groups: the blank group, experimental group, and positive group, each consisting of 10 mice, and the negative group that was composed of 20 mice. The mice in the blank group (group C) were force-fed with normal saline and low-fat feed, the experimental group (group D) with a solution of the formula powder and high-fat feed, the negative group (group P) with normal saline and high-fat feed, and the positive group (group Y) with Orlistat solution and high-fat feed. Each mouse was administered 0.2 mL of the solution by gavage. During the 9-week experimental period, the mice were reared in the same room in different cages, each containing 3–4 mice, and were provided unrestricted access to drinking water and food. The other conditions were a photoperiod of 12 h, temperature of 23 ± 2 °C, and humidity of 60%.

#### Measurement of Mouse Bodyweight and Sampling

During the 9-week experimental period, the bodyweights of mice were measured twice per week. After the experimental period, the mouse liver and colon contents were collected after fasting for 12 h and were kept in the refrigerator at −80 °C for subsequent intestinal microbiota and enzyme activity tests.

#### Measurement of Mouse Intestinal Microbiota

The total DNA of the microbial community was extracted using the E.Z.N.A.® Soil DNA Kit (Omega Bio-Tek, Norcross, GA, USA) according to the manufacturer's instructions.

The quality of extracted DNA was checked using 1% agarose gel electrophoresis, while the concentration and purity of DNA were measured using a NanoDrop2000 Spectrophotometer. The V3–V4 variable regions of the 16S rRNA gene were amplified using PCR with the primers 338F (5′-ACTCCTACGGGAGGCAGCAG-3′) and 806R (5′-GGACTACHVGGGTWTCTAAT-3′). The PCR conditions were: pre-denaturation at 95 °C for 3 min, followed by 27 cycles of denaturation at 95 °C for 30s, annealing at 55 °C for 30s, and extension at 72 °C for 30s, and stable extension at 72 °C for 10 min. The amplicons were stored at 4 °C.

The PCR reaction mixture was composed of 5 × TransStartFastPfu buffer (4 μL), 2.5 mM dNTPs (2 μL), 5 μM upstream primer (0.8 μL), 5 μM downstream primer (0.8 μL), TransStartFastPfu DNA polymerase (0.4 μL), template DNA (10 ng), and dd.H_2_O (final volume to 20 μL). Each reaction was repeated thrice.

The PCR products of the same sample were first mixed and then resolved with 2% agarose gel, after which, the recovered products were purified using an AxyPrep DNA Gel Extraction Kit (Axygen Biosciences, CA, USA), checked using 2% agarose gel electrophoresis, and quantified using a Quantus™ Fluorometer (Promega, USA). The database was constructed using a NEXTflexTM Rapid DNA-Seq Kit (Bioo Scientific, USA), and the gene sequencing was performed using a Miseq PE300/NovaSeq PE250 platform (Illumina, USA).

#### Measurement of Enzyme Activity

The leptin content, insulin content, AMPK activity, FAS activity, LPL activity, SREBPs activity, and ACC1 activity were measured using the double-antibody sandwich enzyme-linked immunosorbent assay (ELISA).

#### Data Processing

The tests were repeated five times, and the data were presented as the mean ±standard deviation. The Least-significant difference(LSD) Duncan analysis was performed using the software SPSS25.0, and the diagrams were drawn using software Majorbio and Origin2018.

## Results and Analysis

### Effect of Formula Powder on Mouse Bodyweight

**Figure 1:**
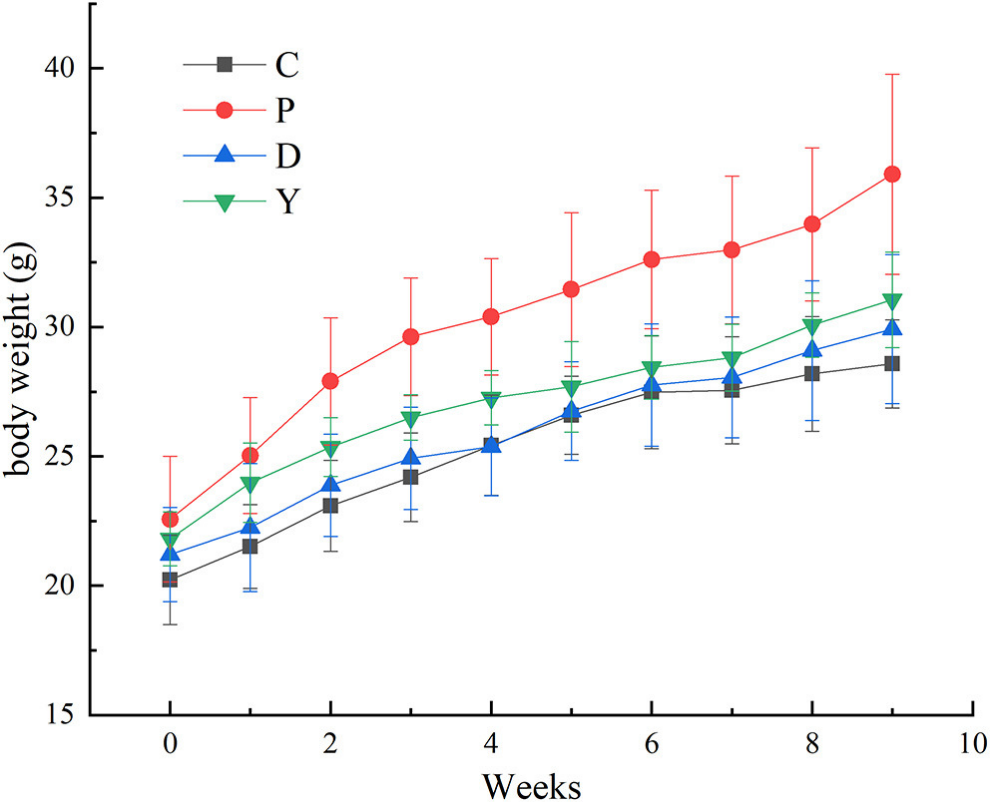
The effect of formula powder on the bodyweight of mice. Group C, mice fed with a standard chow diet; Group P, mice fed with a high-fat diet; Group D, mice fed with a high-fat diet with high-fiber formula powder; Group D, mice fed with a high-fat diet with Orlistat. Values are presented as mean ± SEM. *n* = 10 (Group C, D and Y), *n* = 20 (Group P).

The bodyweights of mice in all four groups showed an increase, which could be attributed to the limited effect of dietary intervention on mouse bodyweight during the growth period (Figure 1). Moreover, the rate of increase in the body weight of mice in group P was higher than that of the remaining three groups, which were similar to each other. This indicated that the formula powder and Orlistat mitigated the excessive increase of mouse bodyweight to attain normal body weight.

### Effect of Formula Powder on the Intestinal Microbiota in the Colon Content of Mice

#### Analysis of α-Diversity Indices of Intestinal Microbiota

The commonly used α-diversity indices included abundance indices (Sobs index and Chao index) and diversity indices (Simpson index and Shannon index) (18, 19). The abundance indices of group C were insignificantly different from those of group D but were significantly different from those of groups P and Y (Table 2). Moreover, the diversity indices of group P were significantly different from those of group C, group D, and group Y, which indicated the similarity between the experimental group and the blank group in terms of species richness and diversity of the intestinal microflora, and the regulatory effect of the formula powder on the intestinal microbiota of mice with a high-fat diet. According to our findings, the prevention of obesity may be achieved by a diet containing high dietary fiber, which regulates the abundance of intestinal microbiota, promotes the process of generation of fermentation metabolites (20), inhibits inflammatory reactions (21), and changes some gene expressions (22).

**Table 2:** Effects of formula powder on the α-diversity of intestinal microflora in mice.

**Group**	**Sobs**	**Shannon**	**Simpson**	**Ace**	**Chao**
Group C	178.33 ± 50.39^b^	2.35 ± 0.69^b^	0.22 ± 0.12^a^	216.25 ± 38.98^b^	216.97 ± 51.72^b^
Group D	202.20 ± 39.14^ab^	2.21 ± 0.51^b^	0.25 ± 0.10^a^	264.78 ± 45.32^ab^	268.69 ± 34.90^ab^
Group P	246.14 ± 36.70^a^	3.46 ± 0.54^a^	0.07 ± 0.04^b^	280.74 ± 48.85^a^	284.57 ± 54.52^a^
Group Y	237.50 ± 43.51^a^	2.62 ± 0.45^b^	0.21 ± 0.08^a^	279.01 ± 30.33^a^	279.18 ± 43.44^a^

*Values are presented as mean ± SEM, n = 5~7. Different lower-case letters in the same column indicate statistically significant differences (P < 0.05) according to Duncan's post-hoc tests*.

#### Analysis of β-Diversity Indices of Intestinal Microbiota

**Figure 2:**
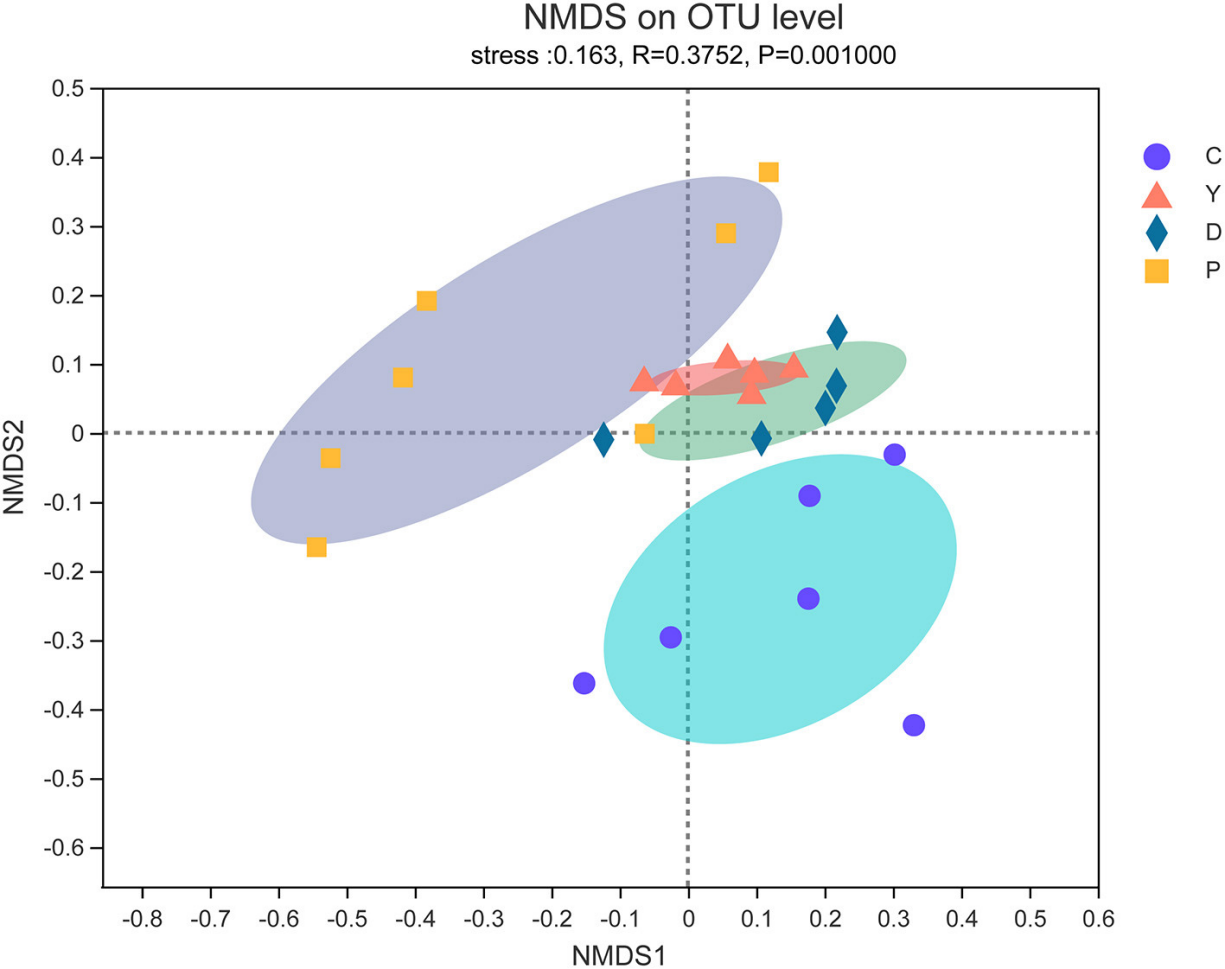
The effect of formula powder on the β-diversity of intestinal microflora in mice. Non-metric multidimensional scaling (NMDS) of weight unifrac distance. Each sample was represented by a dot (*n* = 5–7).

The β-diversity is regarded as the diversity between samples and reflects the differences between samples in community composition (23). In this study, the effect of the formula powder on the community composition of mouse intestinal microflora was analyzed and studied using the non-metric multidimensional scaling (NMDS) method based on weighted unifrac distances. The community compositions of group D and group Y were like those of group P, while that of group D was like that of group C (Figure 2), which also indicates the regulatory effect of the formula powder on mouse intestinal microbiota.

#### Analysis of the Community Composition of Intestinal Microbiota at the Phylum Level

**Figure 3:**
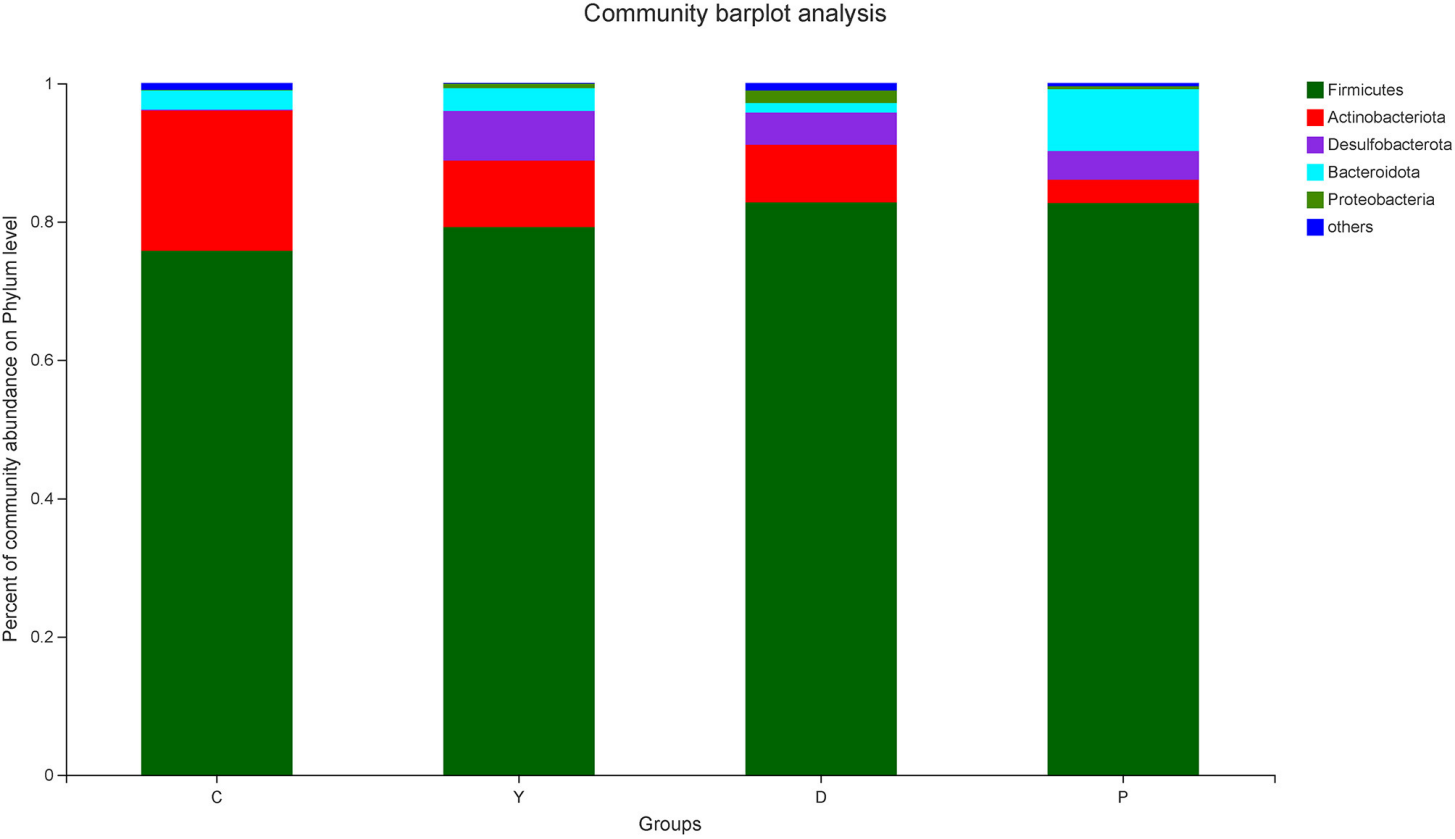
The effect of formula powder on the relative abundance of microorganisms in the colonic contents of mice at the phylum level. Relative abundance of core bacterial families. Kruskal–Wallis rank sum test with Tukey–Kramer *post-hoc* analysis was performed (*n* = 5~7). Same below.

Analysis of the community composition of the mouse intestinal microflora at the phylum level indicated that the three dominant phyla in the colon content of group C were *Firmicutes, Actinobacteria*, and *Bacteroidetds*, while the abundance of *Firmicutes* in group C was lower than that observed in the remaining three groups (Figure 3). A previous study has reported that *Firmicutes* mainly promote the absorption of energy from the diet of their host (24), and obesity is mainly associated with the imbalance of energy uptake, which is associated with an abnormal increase in the proportion of *Firmicutes* and a decrease in *Bacteroides*. In this study, the abundances of various phyla of microorganisms in all four groups were within the normal range, and the relative abundance of *Firmicutes* in group D was high, which could be attributed to the regulation of the pH value of the intestinal tract and an increase in the proportions of *Firmicutes* and *Bacteroides* by dietary fiber, which improved the environment for beneficial bacteria. Previous research has revealed that the abundance of *Firmicutes* in the intestinal tract may be increased by a high resistant-starch diet (25).

#### Analysis of Community Composition of Intestinal Microbiota at the Genus Level

**Figure 4:**
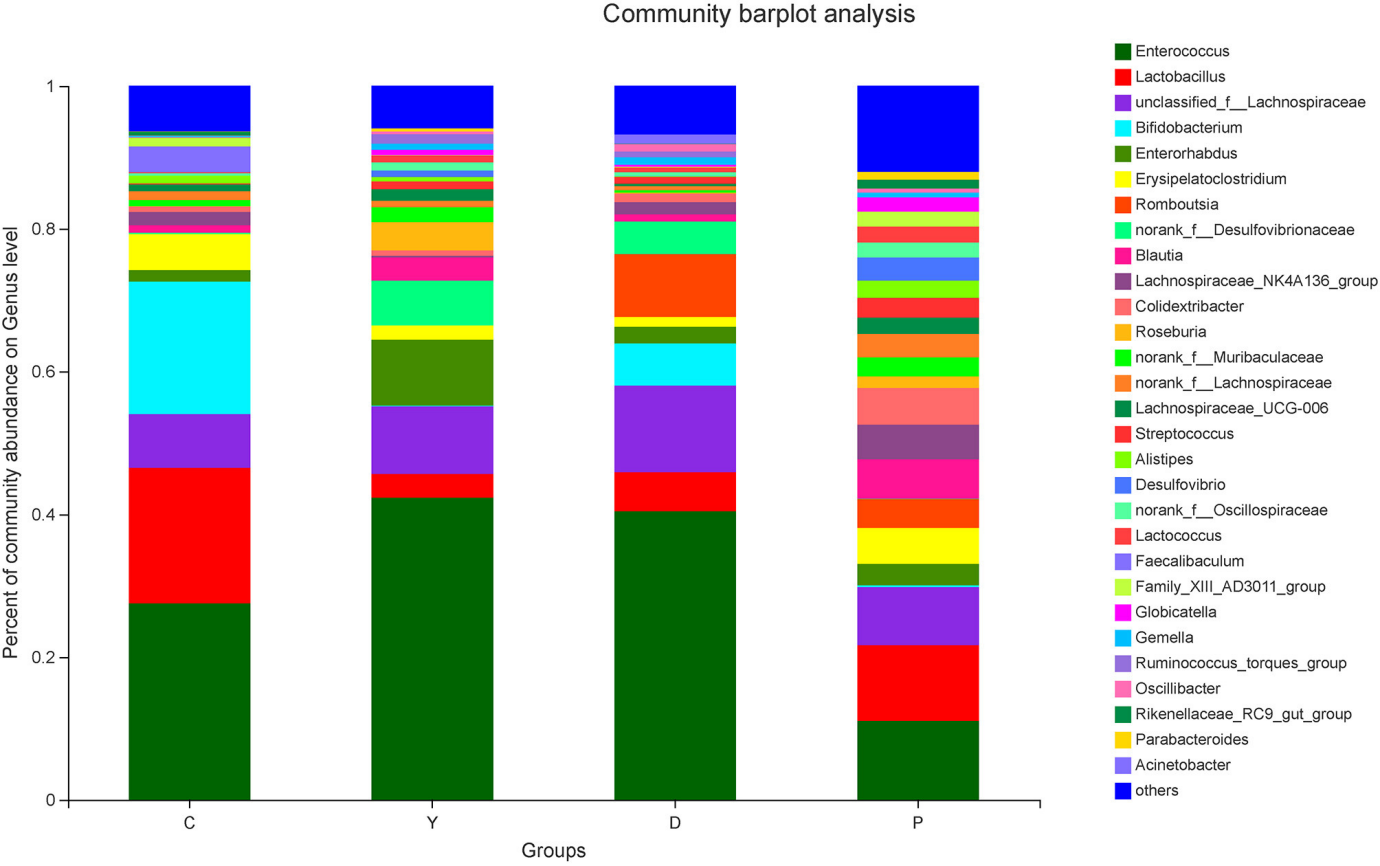
The effect of formula powder on the relative abundance of microorganisms at the genus level in the colonic contents of mice.

**Figure 5:**
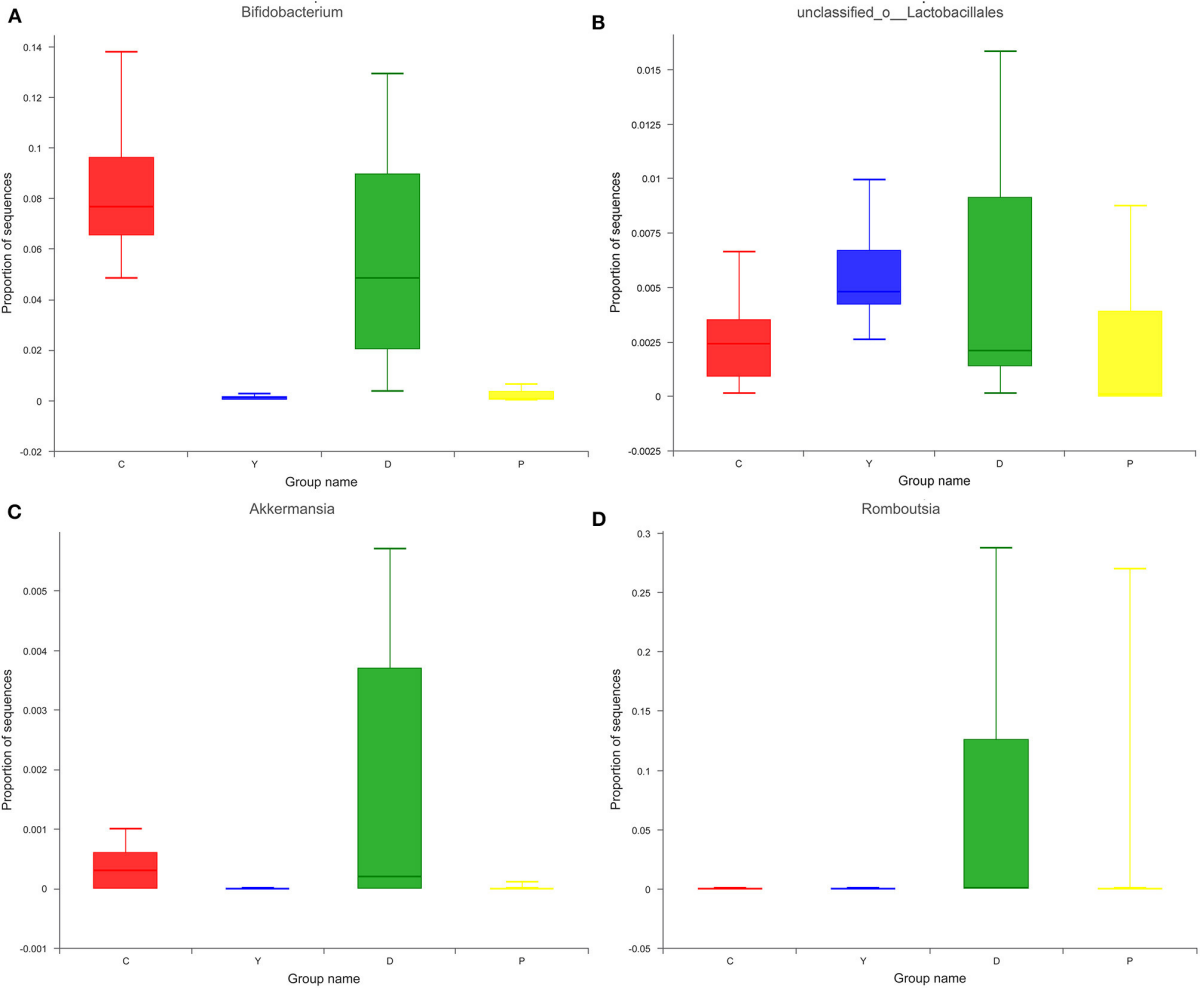
Effects of formula powder on the relative abundance of *Bifidobacterium* **(A)** *unclassified_o_Lactobacillales* **(B)** *Akkermansia* **(C)** and *Romboutsia* **(D)** in mouse colon contents.

The relatively abundant genera in the colon contents of all four groups in descending order were *Enterococcus, Lactobacillus*, unclassified_f_Lachnospiraceae, *Bifidobacterium*, and *Enterorhabdus* (Figure 4). Of these, the relative abundance of *Enterococcus*, which is capable of starch degradation and fermentation of dietary fiber, was the highest in group Y (43%), followed by group D (42%), and the lowest in group P (11%). For *Lactobacillus*, the relative abundance was the highest in group C (19%), followed by group P (11%), and the lowest in group Y (3%). In the case of *Lachnospiraceae*, which can generate short-chain fatty acids, the relative abundance was the highest in group D (12%), followed by group Y (9%), and the lowest in group C (7%). For *Bifidobacterium*, which is a well-known beneficial genus, the relative abundance was the highest in group C (19%), followed by group D (6%), and very low for group P(0.2%) and group Y(0.3%). The intestinal contents of all four groups were characterized by relatively diverse microorganisms at the genus level and were dominated by beneficial bacteria, followed by neutral bacteria (conditionally pathogenic bacteria), and pathogenic bacteria. Previous research has suggested that the presence of all bacteria is important for the dynamic equilibrium of the intestinal tract (26), although the existence of relatively large amounts of beneficial bacteria is indispensable for human health. In this study, the bacteria were screened, and some beneficial bacteria have been listed. The relative abundances of *Bifidobacterium, unclassified_o_Lactobacillales, Akkermansia*, and *Romboutsia* were high in group D (Figure 5), which could be attributed to the presence of large amounts of dietary fiber and resistant starch in the formula powder, the use of extrusion treatment, which combined gelatinized starch and lipids into compound materials, and the introduction of konjac powder, which filled the space between material particles as colloids after hygroscopic expansion to form denser compound materials to provide the properties of RS5 resistant starch (27). Moreover, the nutrition was remarkably improved, which could be attributed to the adoption of super-micro grounding, which converts the insoluble dietary fiber in the oat bran into soluble dietary fiber (28). Previous research has reported that resistant starch can decrease the pH of the fermentation system and promote the generation of lactic acid to provide a favorable environment for the multiplication of beneficial bacteria, while RS5 resistant starch can more effectively promote the multiplication of *Bifidobacterium* and *Romboutsia* than other types of resistant starch (29), which is consistent with the findings of our research. We observed that *Romboutsia* was more abundant in the healthy mucosa of the human body (30). Another study has reported that *Romboutsia* is positively correlated with a lower risk of type-I diabetes in some areas (31).

### Effect of Formula Powder on Leptin Content in Mouse Liver

**Figure 6:**
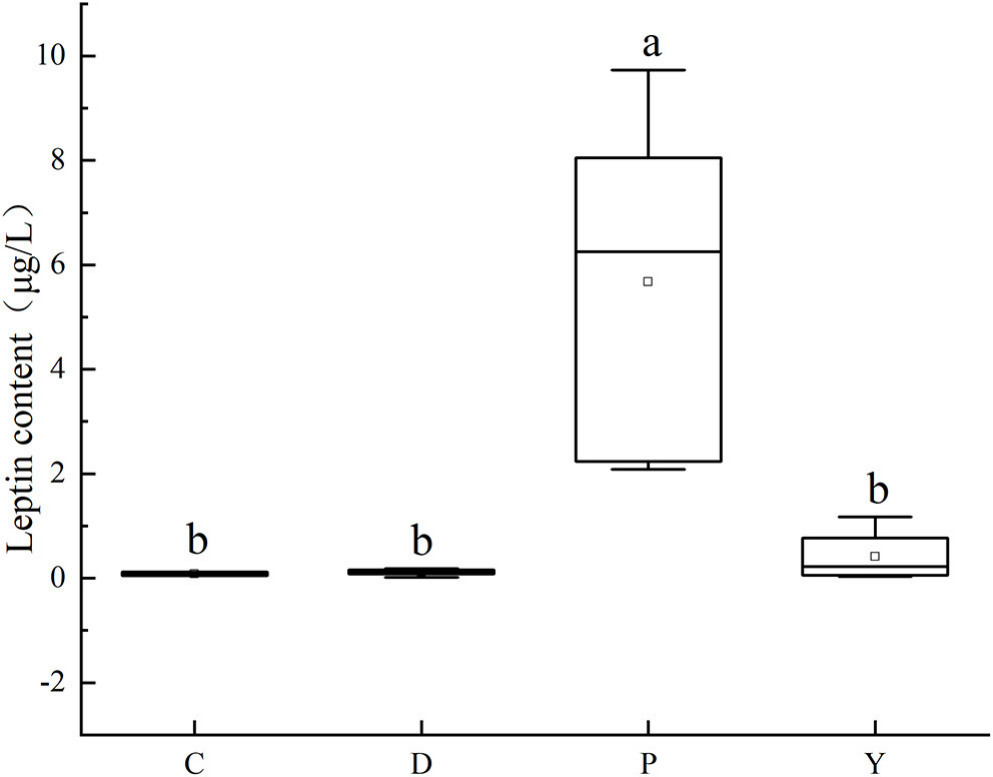
Effect of formula powder on leptin content in mouse liver. a–d The different letters mean that there are significant differences (*p* < 0.05) between every two groups, and the same letters mean that there is no significant difference (*p* > 0.05) between every two groups according to Duncan's multiple range test. *n* =5. Same below.

Leptin is a key factor in the regulation of the energy balance of body cells (32). It was observed that the leptin content of group P was significantly different compared to that of the remaining three groups (*P* < 0.05) (Figure 6), which could be attributed to the occurrence of hyperleptinemia associated with a long-term high-fat diet and the occurrence of leptin resistance like insulin resistance (33, 34). Moreover, the leptin content of group C was low, which could be attributed to the inhibition of abnormal fat accumulation in the mouse liver under the effect of leptin produced by a normal diet on the brain via the brain-vagus-liver axis. Besides, the leptin contents were similar between group D, group Y, and group C (*P* > 0.05), highlighting the promoting effect of the formula powder and weight-loss drug on the normal leptin metabolism and the gradual mitigation of leptin sensitivity of the hypothalamic neurons in fat mice to alleviate the leptin resistance (35).

### Effect of Formula Powder on the Insulin Content in Mouse Liver

**Figure 7:**
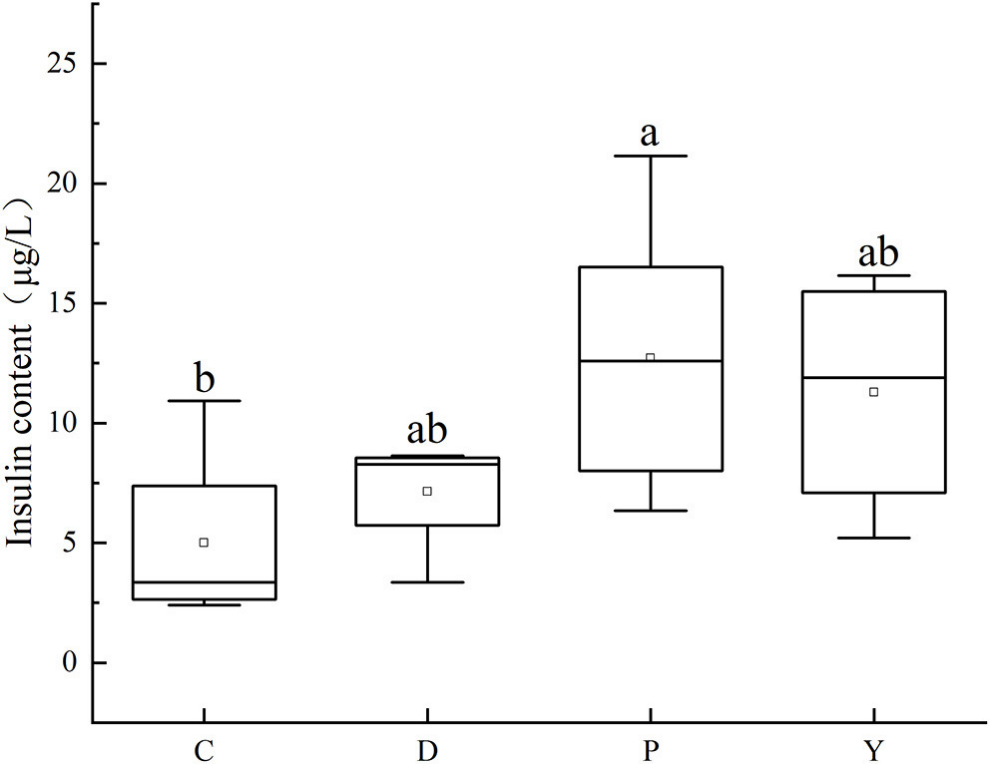
Effects of formula powder on the insulin content in mouse liver. The different letters mean that there are significant differences (*p* < 0.05) between every two groups, and the same letters mean that there is no significant difference (*p* > 0.05) between every two groups according to Duncan's multiple range test. n = 5.

Among the studied groups, the insulin content followed the order group P>group Y>group D>group C, with the insulin content of group C being similar to that of group D (*P* > 0.05) but significantly different from that of group P (*P* < 0.05) (Figure 7). In this study, the insulin content of group P was significantly higher than that of group C, which could be attributed to the occurrence of insulin resistance like leptin resistance and the existence of conditions such as hypercholesterolemia and hyperleptinemia associated with a high-fat high-energy diet (36). Besides, the insulin content of group D was like that of group C, which could be attributed to the effects of the formula powder in alleviating the insulin resistance associated with a high-fat diet and improving blood sugar regulation. Meanwhile, the insulin content of group Y was like that of group P, which could be attributed to side-effects such as diarrhea and blood sugar metabolic disorder associated with the weight loss drug Orlistat.

### Effects of Formula Powder on AMPK, FAS, SREBPs, LPL, and ACC1 Activities in Mouse Liver

AMPK can maintain the energy homeostasis of body cells, including the lipid metabolic balance, and when in an activated state, can decrease lipid synthesis by inhibiting the genetic expressions of downstream enzymes (37), including SREBPs, FAS, and ACC1 (38). The activation or gene expression of these downstream enzymes can increase the synthesis and deposition of fat. Meanwhile, AMPK can increase the oxidation of fatty acids and decomposition of fat as the main upstream conditioning agent of downstream enzymes, including LPL and hepatic lipase (HL). As a rate-limiting enzyme for triglyceride (TG) metabolism, LPL can catalyze the decomposition of TG into fatty acids and monoglycerides, and regulate the exchange between lipoproteins (39), making it an important factor in glycolipid metabolism.

**Figure 8:**
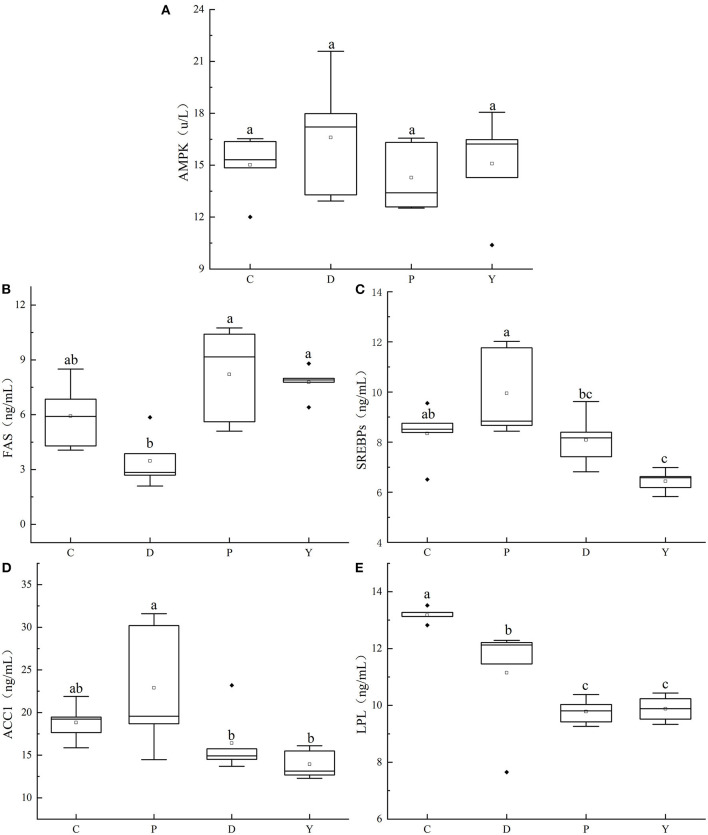
Effects of formula powder on the activities of AMPK **(A)**, FAS **(B)**, SREBPs **(C)**, ACC1 **(D)**, and LPL **(E)** in mouse liver. The different letters mean that there are significant differences (*p* < 0.05) between every two groups, and the same letters mean that there is no significant difference (*p* > 0.05) between every two groups according to Duncan's multiple range test. n = 5.

The effects of the formula powder on the AMPK, FAS, SREBPs, LPL, and ACC1 activities in mouse liver were studied (Figure 8). The AMPK activity followed the pattern of group D > group Y > group C > group P and did not differ significantly between the groups. The LPL activity followed the order of group C > group D > group Y > group P, being significantly higher in group D than group P but significantly lower than group C. Furthermore, the FAS followed the pattern of group P > group Y > group C > group D, being significantly lower in group D than group P but similar to group C. Finally, the activities of SREBPs and ACC1 followed the order of group P > group C > group D > group Y, being significantly lower in group D than group P but similar to group C. These results indicate that the formula powder has a certain regulatory effect on the lipid metabolism disorder caused by high-fat diet. It can activate AMPK, increase LPL enzyme activity, inhibit the enzyme activity of SREBPs, FAS and ACC1, and promptly remove excess cholesterol and triglycerides in the liver. Alleviation of hepatic steatosis caused by a high-fat diet. The regulatory effect of the formula powder is correlated with the presence of large amounts of dietary fiber and micronutrients such as resistant starch and polyphenols, substances that alleviate the liver injuries, inflammation, and disturbance of intestinal microbiota associated with a high-fat diet. According to the research by Christine et al. (40), an increase in the intake of dietary fiber by 5 g per day can decrease the risk of disease progression or mortality by 30% and facilitate immune therapy. Besides, according to the research by Xu et al. (41), oats can reduce total cholesterol content and low-density lipoprotein content and possess the benefits of a prebiotic.

### Analysis of Correlation of Intestinal Microbiota With the Activities of Key Enzymes Involved in Liver Metabolism

**Figure 9:**
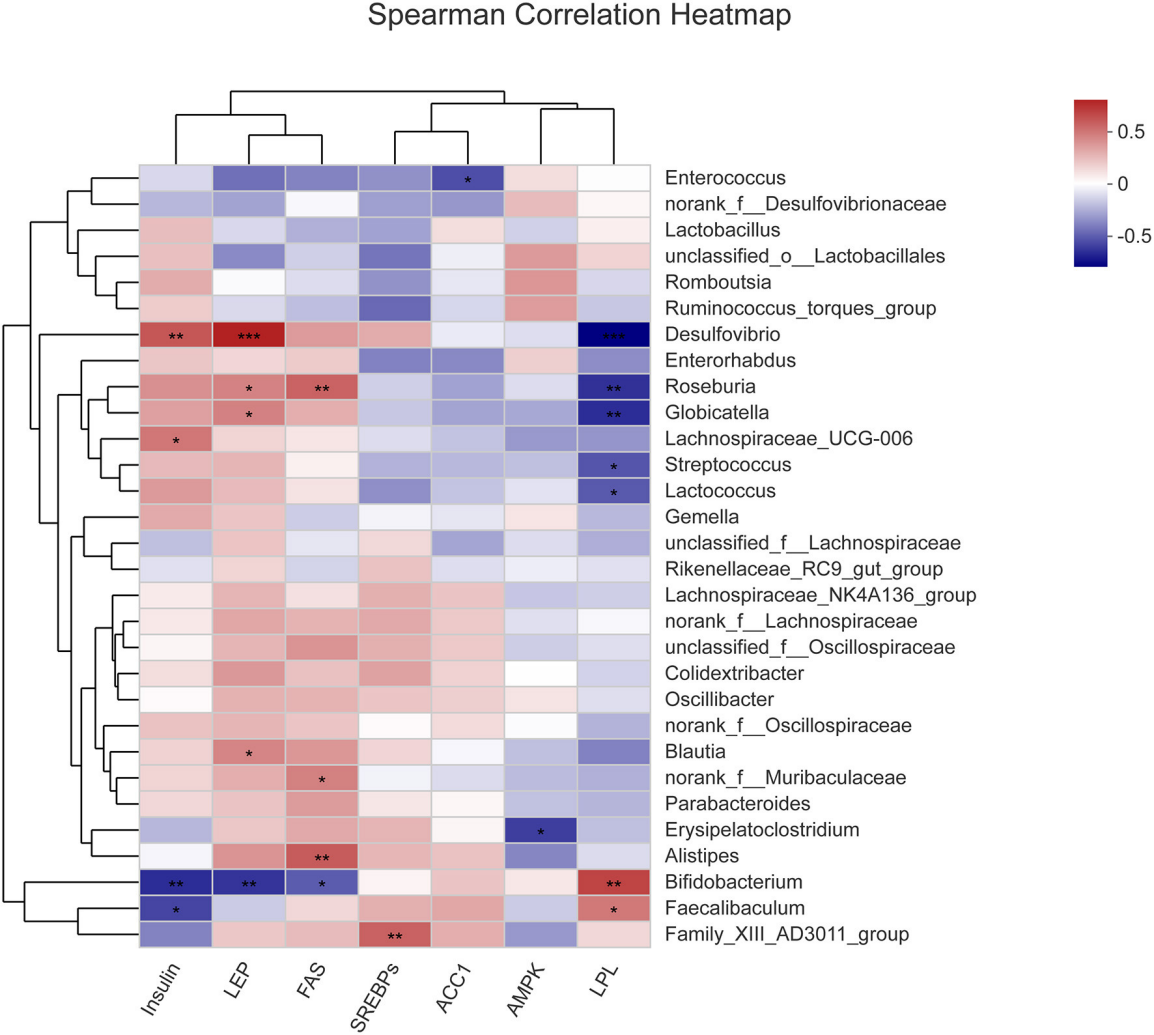
Correlation between gut microbiota and activities of key enzymes in liver metabolism. Group C, mice fed with a standard chow diet; Group P, mice fed with a high-fat diet; Group D, mice fed with a high-fat diet with high-fiber formula powder; Group D, mice fed with a high-fat diet with Orlistat. on all of the Liver enzyme activity, including Insulin, LEP, FAS, SREBPs, ACC1, AMPK, and LPL. Orange represents positive correlation, while blue represents negative correlation. *p < 0.05, **p ≤ 0.01, ***p ≤ 0.001.

The correlation between the abundance of various genera comprising the intestinal microbiota and the activities of relevant enzymes in liver metabolism was analyzed (Figure 9). It was observed that *Desulfovibrio* was highly significantly positively correlated with insulin and LEP (P < 0.01 and P < 0.001) and highly significantly negatively correlated with LPL (P < 0.001). Besides, *Roseburia* was significantly positively correlated with LEP and FAS (P < 0.05 and P < 0.01) and significantly negatively correlated with LPL (P < 0.01). Moreover, *Globicatella* was significantly positively correlated with LEP (*P* < 0.05) and significantly negatively correlated with LPL (*P* < 0.01). Furthermore, *ErysiPelatoclostridium* was significantly negatively correlated with AMPK (*P* < 0.05), while *Alistipes* was significantly positively correlated with FAS (*P* < 0.01). Also, *Bifidobacterium* was highly significantly negatively correlated with insulin and LEP (*P* < 0.01), significantly negatively correlated with FAS (*P* < 0.05), and significantly positively correlated with LPL (*P* < 0.01). Finally, *Faecalibaculum* was significantly positively correlated with insulin (*P* < 0.05) and significantly negatively correlated with LPL (*P* < 0.05). The results of correlation analysis indicate that some pathogenic bacteria were positively correlated with LEP, FAS, and insulin and negatively correlated with LPL, while some beneficial bacteria were positively correlated with LPL.

## Conclusion

The formula powder prepared using extrusion treatment, and super micro grinding was rich in dietary fiber and diverse bio-active ingredients and could provide long-term satiety and reduce energy intake. Our results suggested that the proposed formula powder could reduce the abnormal increase in mouse body weight associated with a long-term high-fat diet during the growth period, while bringing the α- and β-diversity of the intestinal microbiota of test mice close to that of normal mice. Besides, the formula powder could regulate the pH of the colon and increase the proportions of the phyla *Firmicutes* and *Bacteroides* to improve the environment for beneficial bacteria. Moreover, the formula powder could promote the normal metabolism of leptin and insulin, alleviate the resistance to leptin and insulin associated with a high-fat diet, and increase the activities of key enzymes in liver metabolism, such as AMPK and LPL, while decreasing the activities of FAS, SREBPs, and ACC1, bringing them to the levels observed in normal mice. The results of the correlation analysis suggested that an increase in liver LPL activity and a decrease in LEP, FAS, and insulin activities could increase the abundance of beneficial bacteria such as *Bifidobacterium* and decrease the abundance of pathogenic bacteria such as *Desulfovibrio*. In conclusion, the proposed formula powder has preventive and mitigating effects on the disturbance of intestinal microbiota and abnormal liver metabolism in mice with a high-fat diet. This study combined extrusion and ultrafine grinding technology to improve the utilization of oat bran, and these processing methods are simple and can replace the use of additives. The research results provide a reference for new functional foods in the field of healthy weight loss.

## Data Availability Statement

The datasets presented in this study can be found in online repositories. The names of the repository/repositories and accession number(s) can be found below: NCBI: PRJNA808203.

## Ethics Statement

The animal study was reviewed and approved by Inner Mongolia Agricultural University Laboratory Animal Welfare and Animal Experimental Ethical Inspection Committee.

## Author Contributions

RH was responsible for designing the experiments, drafted the manuscript, conduct animal experiments, and data analysis. YaZ helped data analysis and experimental design. XB was responsible for Organized and collected experimental materials. YuZ and XG was responsible for supervising the animal experiment and experimental activity. MZ handled the supervision throughout research and manuscript publishing. All authors have read and approved the final manuscript.

## Funding

This work was supported by key project of Science and Technology Department of Inner Mongolia (2020GG0064), project of National Natural Science Foundation (32060515), and Major Project of Inner Mongolia Science and Technology Department (2021SZD0017).

## Conflict of Interest

The authors declare that the research was conducted in the absence of any commercial or financial relationships that could be construed as a potential conflict of interest.

## Publisher's Note

All claims expressed in this article are solely those of the authors and do not necessarily represent those of their affiliated organizations, or those of the publisher, the editors and the reviewers. Any product that may be evaluated in this article, or claim that may be made by its manufacturer, is not guaranteed or endorsed by the publisher.
